# Supplementary figures and images for: Understanding allergic multimorbidity within the non-eosinophilic interactome
Source: PLoS One. 2019 Nov 6;14(11):e0224448. doi: 10.1371/journal.pone.0224448 (PMC6834334; doi:10.1371/journal.pone.0224448)

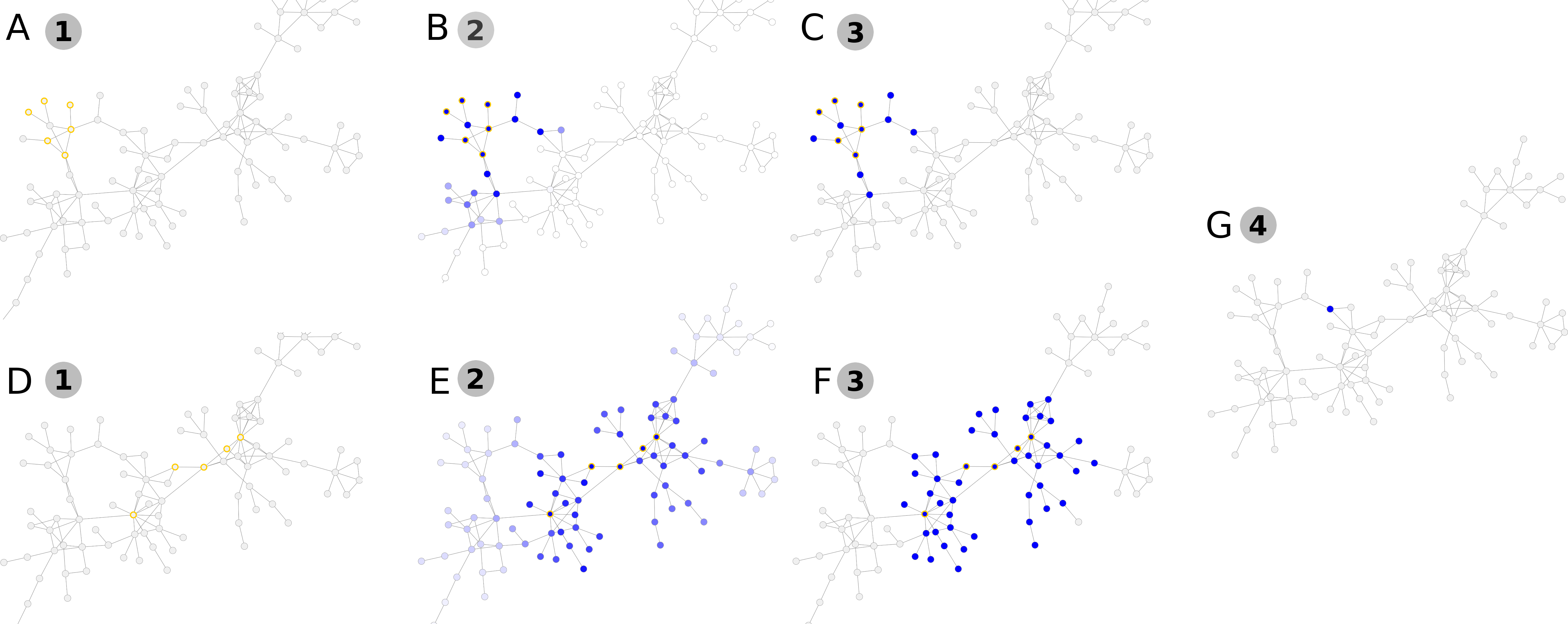

Supplement: S1 Fig — This toy example uses a simplified network of the cell type c, where we will measure the multimorbidity score MS for diseases d1 and d2. The numbers circled in grey correspond to the numbered steps in the section Calculating cell-type-specific multimorbidity of Methods. (A) Genes associated to dis1 (6, orange border) are given an initial score of 1, while all other genes are given a score of 0. (B) The NetScore algorithm scores all genes in the network according to their connectivity to the D-associated genes (blue gradient). Genes in closer proximity to dis1-associated genes get higher scores. (C) The top-scoring genes are selected (in blue). Disease dis1 has 13 top-scoring genes (Scdis1). (D) Genes associated to dis2 (5, in orange border) are given an initial score of 1, while all other genes are given a score of 0. (E) The NetScore algorithm scores all genes according to their connectivity to the dis2-associated genes (blue gradient). (F) The top-scoring genes are selected (in blue). Disease dis2 has 47 top-scoring genes (Scdis2). (G) There is 1 gene common to both top-scoring sets (in blue). The Multimorbidity Score (MS) of the diseases is calculated as the Sorensen-Dice overlap between their top-scoring gene sets. In this case, MScdis1,dis2 is (2 · 1) / (6 + 47) = 0.038. A permutation test over 103 iterations will establish if MScdis1,dis2 is statistically significant (P < 0.05). (PNG) [file pone.0224448.s001.png]

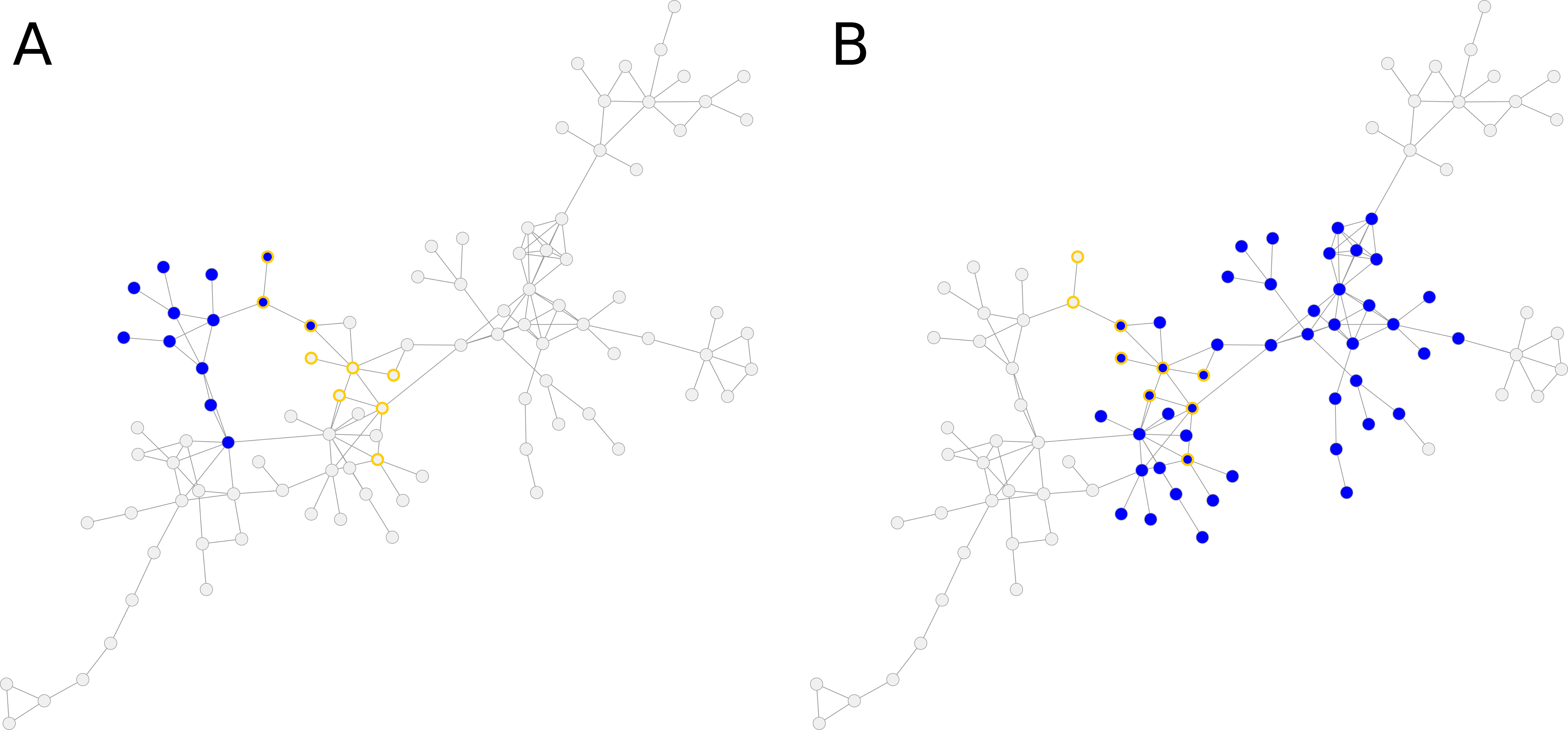

Supplement: S2 Fig — This example uses the network of S1 Fig (225 genes). The pathway P has a total of annotated 20 genes, of which 9 are in the network (shown in orange border). (A) The 13 top-scoring genes for disease d1 (Sc d1; see S1C Fig) are shown in blue, and there are 3 pathway genes within this set. Thus, the perturbation score PSc d1,P is (9/20) / (13/225) = 7.79. For the sake of the example, we will assume that this value is significantly larger than random expectation (P < 0.05). (B) The 47 top-scoring genes for disease d2 (S cd2; see S1F Fig) are shown in blue. There are 7 pathway genes within the Scd2 set. Thus, the perturbation score PScd2,P is (9/20) / (47/225) = 2.15. For the sake of the example, we will assume that this value is significantly larger than random expectation as well (P < 0.05). Consequently, because pathway P is significantly associated to (or perturbed by) diseases d1 and d2, we assume that it is part of the mechanism of multimorbidity between dis1 and dis2 in cell type c. (PNG) [file pone.0224448.s002.png]
