# Supplementary material for: Understanding allergic multimorbidity within the non-eosinophilic interactome
Source: PLoS One. 2019 Nov 6;14(11):e0224448. doi: 10.1371/journal.pone.0224448 (PMC6834334; doi:10.1371/journal.pone.0224448)
Supplement: S1 Text — (PDF) [file pone.0224448.s014.pdf]

Supplementary Methods

Data sources

Gene-disease associations

We built the sets of genes associated to A, D and R by integrating data from four sources: (1) The Comparative Toxicogenomics Database [38], which provides highly reliable gene-disease associations characterized through various experimental procedures combined with a process of expert curation of the literature and other databases (e.g. OMIM [39]). Diseases were identified using the MeSH terms D001249 for A, D003876 for D and D065631 for R. Only genes labeled as *marker* (i.e. experimentally associated with a disease) were considered. (2) The DisGeNet catalog, that contains curated gene-disease associations extracted from literature (for reliability, only gene-disease associations with DisGeNet score > 0.2 were considered) [40]. (3) UniProt-derived gene-disease associations, extracted from the *Involvement in disease* section of the Uniprot Knowledgebase (which contains curated literature-based information as well as information derived from OMIM database [41]). (4) The Phenotype-Genotype Integrator database, that integrates information various NCBI genomic databases with association data from the National Human Genome Research Institute GWAS Catalog [42]. This is the only data source containing solely GWAS-derived gene associations. Only associations with a p-value $< 5 \cdot 10^{-8}$ were considered [43]. Genes associated to a disease d (any of A, D or R) will be hereinafter referred to as d-associated genes.

The interactome

We built the functional interaction network (hereinafter called the *interactome* for brevity) by combining data from: (1) The Reactome Functional Interaction Network (v. 022717) [44], which includes not only protein-protein interactions but also gene expression interaction, metabolic interactions and signal transduction. Interactions annotated as *predicted* were discarded, as were those with score ≤ 0.5 as suggested by the authors. (2) The STRING interaction network (v.10.5), considering only those interactions with score > 700 (high confidence interactions) [45].

Cell-type-specific gene expression

Gene expression levels were obtained from the human gene expression atlas available at ArrayExpress under accession number E-MTAB-62 [46]. This is a cell-type-wide compendium of high-quality microarray-derived expression data that has been previously used in other network-based analysis of gene expression [47-49] and has been incorporated into a number of biomedical software packages [50-52]. In order to maximize consistency and avoid noise in the gene expression levels, only adult human samples and cell types not subjected to any treatments (e.g. treated with DMSO) neither exposed to particular environmental factors (e.g. tobacco smoke, UV) were considered. Consequently, gene expression levels correspond to human subjects in normal healthy state. We removed redundancies in the data source (e.g. *lymphocyte* was found both as a cell type and as a tissue; we only considered the former). We also removed the cell type named *peripheral blood leukocytes* and *peripheral blood mononuclear cells* because they contained a mixture of cell types including eosinophils. We centered and standardized the expression level of each gene as:

$$e_{g,c} = \frac{\left(E_{g,c} - M_g\right)}{MAD_g}$$

where $E_{g,t}$ is the expression level of the gene *g* in cell type *c*, M_g is the median expression level the gene *g* across all cell types, and MAD_g is the median absolute deviation of the expression levels of gene *g* across all cell types. This made the expression levels comparable between genes [53, 54]. We defined a gene to be cell-type-specific if its absolute normalized expression level $e_{g,t}$ was at least 1.5 larger than the interquartile range (IQR) of its normalized expression across all cell types [6, 12, 55, 56]. Genes specific to a cell type *c* (any of our cell types of interest) will be hereinafter referred to as *c*-specific genes.

Cellular pathways

Cellular pathways were downloaded from Reactome database in the *UniProt2Reactome* format files [44]. Pathway-associated genes without expression data or not in the interactome were not considered. Disease-related cellular pathways (e.g. *Constitutive Signaling by Aberrant PI3K in Cancer*) were not considered. Reactome is a collection of pathways built in a hierarchical manner, where larger pathways are subdivided into smaller pathways with more specific functionalities. This implies a trade-off between the specificity in the representation of cellular functions and the average number of genes per pathway [57]. To minimize the overlap between pathways in order to avoid redundancies that could negatively affect our analysis [58], we calculated the pairwise overlap between pathways at distinct levels of the Reactome hierarchy using the Sorensen-Dice method [59-61]. If two pathways had an overlap of > 50% genes, the one with the lowest number of associated genes was removed from the set. We chose pathways of at depth 3 of the hierarchy because it provided a mean overlap < 1% while annotating 4,809 genes (this is 87,9 % of the total genes annotated in the database, all levels considered). Genes associated to a pathway *p* (any of our pathways of interest) will be hereinafter referred to as *p*-associated genes.
Pathway annotation in our previous study of A, D and R were extracted from BioCarta [62]. There is not a perfect equivalence between cellular pathways from BioCarta and Reactome databases, so in order to compare our results to those from our previous whole-organism multimorbidity study [21] we performed an association test to identify which BioCarta pathways significantly overlapped with Reactome pathways (Fisher's Exact test, adjusted P <0.05; **S1 Table**).

Cell-type-specific networks

In order to generate the cell-type-specific network for any cell type *c*, we selected all edges from the interactome connecting *c*-specific genes [6, 63]. This implies that any *c*-specific gene not connected to at least another *c*-specific gene was not considered part of the *c*-specific network. Consequently, *c*-specific networks may contain a subset of all *c*-specific genes. Because of the interactome-based nature of our analysis, those genes not present in the interactome or not present in the expression dataset were removed from the analysis. The statistical significance of the number of *d*-associated genes present in each *c*-specific network was calculated by means of a Fisher's Exact test (adjusted P <0.05, adjusted by the Benjamini-Hochberg method for false discovery (FDR) control [64]).

Quantifying cell-type-specific multimorbidity

In order to obtain a quantitative measure of the extent to which A, D and R multimorbidity is manifested in distinct cell types, we designed an interactome-based approach (workflow in **Figure 1**; illustrated with an example in **S1 Figure**). Briefly, we scored all genes specific to a given cell type according to their connectivity (or their "closeness") to known disease-associated genes, under the rationale that the malfunction of one (or more) of the disease-associated genes is likely to perturb the function of the neighboring genes, eventually disrupting a cellular mechanism and giving rise to a diseased phenotype [5, 65-68]. In other words, we scored each gene in each cell type according to its contribution to the manifestation of A, D and R. Then, we selected the set of top-scoring genes (called *S*; S^c_d being the top-scoring genes for disease *d* in cell type *c*). Finally, for each cell type we calculated the overlap between the sets of top-scoring genes for AD, AR, DR and ADR.

This overlap was called the Multimorbidity Score (*MS; $MS^T_{d1,d2}$* being the Multimorbidity Score for diseases *d1* and *d2* in cell type *c*). The process consisted in the following 4 steps:

• **Step 1: initialization of the network**. *D*-associated genes were mapped onto the *c*-specific network. All genes in the network were initially scored to 0 except for the *d*-associated genes, which were scored to 1.

• **Step 2: gene scoring**. The score of all the genes in the network was updated using the *NetScore* algorithm (as implemented in the GUILD software package with default parameters [67]). *NetScore* belongs to the guilt-by-association family of algorithms, meaning that it calculates the scores of the genes by propagating information from a source gene to a target gene considering the number of shortest paths between them. Consequently, genes with a higher final score will be those more connected to (or "closer" to) the target genes (although they might not be *directly* connected to any target genes). *NetScore* has proven to be robust to incompleteness in the interactome data [68]. In our case, the *NetScore* scored all genes in a cell-type-specific network *c* within the 0-1 range according to their connectivity to the *d*-associated genes (where genes with scores closer to 1 are those more susceptible to be perturbed by disease *d* in cell type *c*). The scores were then transformed into *z*-scores [20], $z^{g,c}_d$ representing the *z*-score of gene *g* for disease *d* in cell type *c*. Simply put, *z* is a numerical quantification of how connected gene *g* is to genes associated to disease *d* in cell type *c* (the more connected it is, more more susceptible it will be by the manifestation of the disease).

• **Step 3: selection of top-scoring genes**. The top-scoring genes were selected by ranking them according to their *z*-score and selecting those with $z >= 1.64$ [20]. We called this set of genes S^c_d.

• **Step 4: calculation of the Multimorbidity Score (*MS*).** *MS* between the two diseases d_1 and d_2 in the cell-type-specific network *c* were obtained by calculating the overlap between sets S^c_{d1} and S^c_{d2} using the Sorensen-Dice similarity index as follows:

$$MS^c_{d1,d2} = \frac{2 \cdot |S^c_{d1} \cap S^c_{d2}|}{\left(|S^T_{d1}| + |S^c_{d2}|\right)}$$

where d_1 and d_2 were the two diseases being compared, and $|S^T_{d1}|$ and $|S^T_{d2}|$ are the number of genes in S^t_{d1} and S^T_{d2}. The generalization of the *MS* formula for the multimorbidity between the three diseases in our set (A, D and R) was calculated as:

$$MS^c_{ADR} = \frac{3 \cdot |S^c_A \cap S^c_D \cap S^c_R|}{\left(|S^c_A| + |S^c_D| + |S^c_R|\right)}$$

Characterizing cell-type-specific multimorbidity mechanisms

After having quantitatively scored the multimorbidity between diseases in different cell types, we wished to identify the actual cellular mechanisms involved in the manifestation of the multimorbidities. To do so, we designed a method to measure the perturbation that a disease can exert over a cellular pathway in a given cell type. The starting point is the set of top-scoring genes S^c_d calculated in the previous section. We identified the set of cellular pathways present in cell type *c*, and then scored how perturbed they were by the manifestation of disease *d* using S^c_d (workflow in **Figure 2**; illustrated with an example in **S2 Figure**). This score was called the Perturbation Score (*PS; $PS^c_{p,d}$* being the perturbation experimented by pathway *p* during the manifestation of disease *d* on cell type *c*). Under the assumption that any disease can be viewed as the product of perturbed cellular mechanisms (i.e. cellular pathways), and that multimorbidity is known to arise as those perturbed mechanisms are shared by distinct diseases [12, 13, 69, 70], we selected as candidate mechanisms for multimorbidity those pathways that were significantly perturbed in more than one disease in the same cell type. The process consisted in the following 7 steps:

• **Step 1**: **initialization of the network.** See Step 1 in the previous section.
• Step 2: gene scoring. See Step 2 in the previous section.
• Step 3: selection of top-scoring genes. See Step 3 in the previous section.
• **Step 4: selection of pathways.** We filtered out unrepresented or poorly connected pathways in *c*. To do so, we devised the connectivity parameter C^c_p, which allowed us measure how interconnected *p*-associated genes were to one another in *c*:

$$C^c_p = 1 - \left[\frac{N^c_p - 1}{n^c_p - 1}\right]$$

where N^c_p is the number of components that *p*-associated genes form in *c*, and n^c_p is the number of *p*-associated genes in *c*. In short, C^T_p tells us to which extent a *p*-associated gene can be reached by another *p*-associated gene in *c*. It will be 0 if all *p*-associated genes are disconnected from one another in *c*, and 1 if there is at least one path connecting any two *p*-associated genes. The rationale behind this parameter is that *p*-associated genes must have a certain connectivity in *c* order to carry out the function described by *p*. For statistical validation, all *c* genes were binned according to the formula *round(log(k)+1)*, where *k* is the number of neighbors of a gene [70]. Then, we built a null model by randomly selecting n_p genes from *c* from the same connectivity bins than the original n_p *p*-associated genes. This ensured that the original connectivity of *p*-associated genes was preserved in the null model. Next, we applied the steps 1 to 4 to the null model and obtained $Crand^c_p$. The procedure was repeated 10^3 times to obtain the null distribution $P(Crand^c_p)$, which allowed us to calculate the probability *P* of observing $Crand^T_p < C^c_p$ over $P(Crand^c_p)$ by means of a *z*-test. Those pathways with a C^c_p larger than random expectation ($P < 0.05$) were selected. All other pathways were considered as not present in *c* and were discarded. As an additional filtering step, we removed those pathways whose largest component had < 3 genes (leaving only largest components with at least 2 edges) [21].
• **Step 5: calculation of Perturbation Score (*PS*)**. For a pathway *p* and a disease *d* in a *c*-specific network, we calculated the perturbation score (PS^c_{pd}) as the ratio of the fraction of *p*-associated genes perturbed by disease *d* in *c* divided by the fraction of *c* genes perturbed by disease *d*:

$$PS^c_{p,d} = \frac{\left[\frac{S^c_p \cap S^c_d}{S^c_p}\right]}{\left[\frac{S^c_d}{S^c}\right]}$$

where S^c_d was calculated in Step 3 of the section *Quantifying cell-type-specific multimorbidity*, S^c_p is the set of *p*-associated genes in cell type *c*, and S^c is the total set of genes of the *c*-specific network. The larger the perturbation score PS^c_{pd}, the more perturbed pathway *p* will be in cell type *c* during the manifestation of disease *d*.
• Step 6: statistical validation. Statistical validation was similar to that carried out in the previous section: a null model was built by randomly selecting n_d genes from *c* from the same connectivity bins than the original n_d *d*-associated genes. Next, we calculated $PSrand^c_{pd}$. The procedure was repeated 10^3 times to obtain the null distribution $P(PSrand^c_{p,d})$, which allowed us to calculate the probability *P* of observing $PSrand^c_{p,d} < PS^c_{p,d}$ over $P(PSrand^c_{p,d})$ by means of a *z*-test. The Benjamini-Hochberg method was used to adjust for multiple testing. Those pathways with a $PS^c_{p,d}$ larger than random expectation ($P < 0.05$) were selected. We called this set of pathways P^c_d.

• **Step 7: identification of mechanisms of multimorbidity.** Pathways associated to multimorbidity between diseases *d1* and *d2* in the *c*-specific network were identified as those common to sets S^c_{d1} and S^c_{d2}. Similarly, candidate pathways for multimorbidity between A, D and R in *c* were identified as those common to sets S^c_A, S^c_D and S^c_R.

Identifying cell-type-specific candidates to multimorbidity

Lastly, we wished to identify individual genes that might constitute candidates to multimorbidity. In the *Quantifying cell-type-specific multimorbidity* section we had identified the sets of genes more susceptible to be perturbed by a disease in a cell type (S^c_d). We identified as multimorbidity candidates those genes simultaneously belonging to >=2 of those sets (i.e. susceptible to be perturbed by two diseases in the same cell type) for AD, AR, DR multimorbidities, and >=3 in the case of ADR multimorbidity. In addition, we numerically scored the contribution of each gene *g* to multimorbidity ($MS^{g,c}_{d1,d2}$ being the Multimorbidity Score for gene *c* with respect to diseases *d1* and *d2* in cell type *c*):

$$MS^{g,c}_{d1,d2} = \frac{z^{g,c}_{d1} + z^{g,c}_{d2}}{2}$$

where d_1 and d_2 were the two diseases of interest, *g* is the gene, and *c* is the cell type. The generalization of the formula for the multimorbidity between the three diseases is:

$$MS^{g,c}_{ADR} = \frac{z^{g,c}_{A} + z^{g,c}_{D} + z^{g,c}_{R}}{3}$$
